# The development of high resolution maps of tsetse abundance to guide interventions against human African trypanosomiasis in northern Uganda

**DOI:** 10.1186/s13071-018-2922-5

**Published:** 2018-06-08

**Authors:** Michelle C. Stanton, Johan Esterhuizen, Inaki Tirados, Hannah Betts, Steve J. Torr

**Affiliations:** 1 Lancaster Medical School, Lancaster University, Lancaster, UK; 2 Vector Biology Department, Liverpool School of Tropical Medicine, Liverpool, UK; 3 Parasitology Department, Liverpool School of Tropical Medicine, Liverpool, UK

**Keywords:** Human African trypanosomiasis, Tsetse flies, Vector control, Geostatistics, Uganda

## Abstract

**Background:**

Vector control is emerging as an important component of global efforts to control Gambian sleeping sickness (human African trypanosomiasis, HAT). The deployment of insecticide-treated targets (“Tiny Targets”) to attract and kill riverine tsetse, the vectors of *Trypanosoma brucei gambiense*, has been shown to be particularly cost-effective. As this method of vector control continues to be implemented across larger areas, knowledge of the abundance of tsetse to guide the deployment of “Tiny Targets” will be of increasing value. In this paper, we use a geostatistical modelling framework to produce maps of estimated tsetse abundance under two scenarios: (i) when accurate data on the local river network are available; and (ii) when river information is sparse.

**Methods:**

Tsetse abundance data were obtained from a pre-intervention survey conducted in northern Uganda in 2010. River network data obtained from either digitised maps or derived from 30 m resolution digital elevation model (DEM) data as a proxy for ground truth data. Other environmental variables were derived from publicly-available resolution remotely sensed data (e.g. Landsat, 30 m resolution). Zero-inflated negative binomial geostatistical models were fitted to the abundance data using an integrated nested Laplace approximation approach, and maps of estimated tsetse abundance were produced.

**Results:**

Restricting the analysis to traps located within 100 m of any river, positive associations were identified between the length of river and the minimum soil/vegetation moisture content of the surrounding area and daily fly catches, whereas negative associations were identified with elevation and distance to the river. The resulting models could accurately distinguish between traps with high and low fly catches (e.g. < 5 or > 5 flies/day), with a ROC-AUC (receiver-operating characteristic - area under the curve) greater than 0.9. Whilst the precise course of the river was not well approximated using the DEM data, the models fitted using DEM-derived river data performed similarly to those that incorporated the more accurate local river information.

**Conclusions:**

These models can now be used to assist in the design, implementation and monitoring of tsetse control operations in northern Uganda and further can be used as a framework by which to undertake similar studies in other areas where *Glossina fuscipes fuscipes* spreads Gambian sleeping sickness.

**Electronic supplementary material:**

The online version of this article (10.1186/s13071-018-2922-5) contains supplementary material, which is available to authorized users.

## Background

Human African Trypanosomiasis (HAT), a severe neglected tropical disease (NTD) affecting communities in sub-Saharan Africa, is caused by two parasites, *Trypanosoma brucei gambiense* and *T*. *b*. *rhodesiense* which are transmitted to humans by the bite of tsetse flies (*Glossina* spp.). The two forms of the disease have distinct, non-overlapping geographical distributions, with Uganda being the only country where both forms exist [1, 2]. The disease caused by *T. b. gambiense*, commonly referred to as Gambian sleeping sickness, is generally considered to be an anthroponosis. The disease, transmitted primarily by riverine tsetse, threatens populations in central and western parts of sub-Saharan Africa, and is responsible for 98% (21,862/22,300) of recent (2012–2016) HAT cases [3]. The second form of the disease, caused by *T. b. rhodesiense,* commonly called Rhodesian sleeping sickness, is a zoonosis which is primarily transmitted by savanna tsetse and is found in east and southern Africa. Within these geographical areas there are clusters or ‘foci’ of HAT transmission, with approximately 360 foci being identified with varying levels of transmission intensity across 36 countries (~300 Gambian, ~60 Rhodesian) [1]. The environmental conditions within these foci vary but tend to be remote rural areas where human populations live close to habitats suitable for tsetse.

There are two approaches to disease control recommended by the World Health Organization: the treatment of cases detected using active and passive surveillance, and vector control. Until recently, it was widely acknowledged that whilst vector control was effective for Rhodesian sleeping sickness through techniques such as treating livestock reservoir hosts with insecticides, vector control for Gambian sleeping sickness was considered infeasible at large geographical scale largely due to the costs associated with available methods of vector control [4, 5]. In response to this, cheaper methods of controlling the vectors of *T. b. gambiense* have been developed in the form of “Tiny Targets”. These consist of a square panel (25 × 25 cm) of blue cloth flanked by an identically-sized piece of black netting both impregnated with insecticide. The targets are deployed along the edges of rivers and lakes in areas where riverine tsetse concentrate. Studies conducted in northern Uganda have shown that the use of “Tiny Targets” is highly cost-effective in comparison to older methods of vector control [4, 6].

As a result of intensified efforts to control and ultimately eliminate Gambian HAT, the number of reported cases is currently on the decline, with 2131 reported globally in 2016 [3, 7]. To sustain these efforts, it is important to ensure that the new vector control strategies being adopted are suited to a range of changing environmental and epidemiological situations whilst remaining scalable and cost-effective [8]. “Tiny Targets” are deployed every 50 metres along rivers within an intervention area where either flies have been previously detected, or where the environment is deemed suitable for riverine species of tsetse, e.g. due to the presence of riparian vegetation. Targets are deployed 1–2 times per year and continuous monitoring to quantify the impact on the tsetse population is conducted using traps. Knowledge of the density and distribution of tsetse obtained by pre-intervention surveys guides the distribution of targets and location of monitoring traps and as such, systems to predict the likely fine-scale distribution and abundance of tsetse would improve ability to design and implement tsetse control operations. In the past this was done by using maps to identify rivers and aerial photographs to identify tsetse habitat. In many places however, especially in the remote areas where Gambian HAT occurs, suitable and recent maps and aerial photographs do not exist. The ability to use remotely sensed data to guide tsetse control has long been recognised [9] and has primarily been used to produce maps of tsetse presence as opposed to abundance, with earlier attempts being more focused on producing predicted output at a course spatial resolution over larger geographical areas [10–12]. In this earlier work, the resolution of the predicted output was limited by the scale of the available remotely sensed data, and as such this output was not suitable for guiding local control efforts. However, with data collected from sources such as Landsat (30 m resolution, new image every 8 days), and ASTER (15–90 m resolution, new image every 16 days) becoming freely available, there is the increasing opportunity to derive maps capable of guiding local tsetse control activities [13, 14]. Further, the ability to predict abundance as opposed to presence/absence has previously been limited by the lack of suitable tsetse count data.

The aim of this study was to use a geostatistical modelling framework to explore the relationship between the natural abundance (i.e. prior to large scale tsetse control activities) of the riverine tsetse species *Glossina fuscipes fuscipes*, the most important vector of *T. b. gambiense* in northern Uganda and environmental variables derived from freely available remotely sensed data. A secondary aim was to determine whether river network data derived from remotely sensed digital elevation data served as a suitable proxy to locally verified river network data, as this more detailed information is frequently unavailable in HAT endemic areas.

## Methods

### Study area

**Fig. 1:**
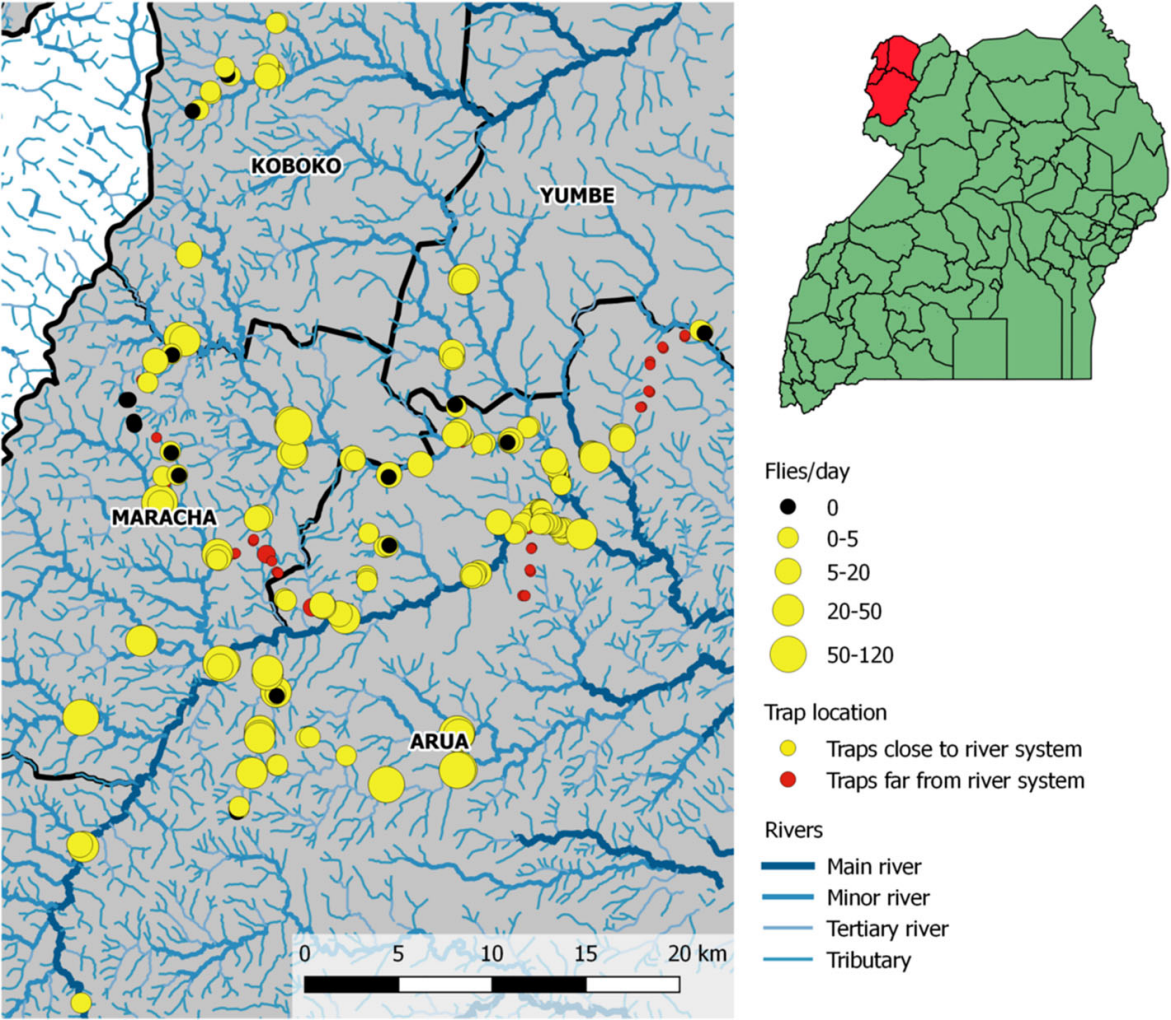
Map depicting the locations of the 236 tsetse traps deployed between October-December 2010 in north west Uganda, and the resulting average daily tsetse count. The location of the surveyed Districts (Arua, Maracha, Koboko and Yumbe) is highlighted in red in the map of Uganda

In north-west Uganda, *G. f. fuscipes* is the vector of *T. b. gambiense*. All field data used in the present study are from Arua, Koboko, Maracha and Yumbe Districts (Fig. 1), a densely settled rural area comprising small-scale farms producing a mixture of subsistence (e.g. cassava, matoke, peanuts) and cash (tobacco) crops and low numbers of livestock (cattle, pigs, goats). There are two large perennial rivers (Enyau, Kochi) which flow into the Nile and large numbers of smaller tributaries and seasonal streams. Narrow (2–5 m) and intermittent bands of natural vegetation (e.g. *Cynometra alexandri*, *Entada abyssinica*, *Acacia seyal*, *Ekebergia capensis*, *Plectranthus barbatus* and *Schrebera alata*) are associated with these rivers and streams. Wild mammalian hosts are rare and the main hosts of tsetse are reptiles (Monitor lizard), cattle, pigs and humans [4].

### Tsetse survey data

A tsetse survey was undertaken in October to December 2010 to provide baseline information on the *G. f. fuscipes* distribution prior to undertaking a “Tiny Target” intervention study [4]. A total of 236 pyramidal traps [15] were deployed across an area of 985 km^2^, and daily tsetse counts were recorded for a period of 1–8 days (median 3 days). These data can be accessed in the Additional file 1. The majority of traps (~90%) were placed along the banks of rivers and streams as this was habitat known to be associated with *G. f*. *fuscipes*. As it was unclear whether flies were likely to be found in areas further from riverine areas, additional traps (~10%) were placed further from the river network in nearby farming areas (Fig. 1).

### Environmental data

Landsat 5 Thematic Mapper (TM) imagery was obtained for the study area for December 2009 as low cloud cover images were unavailable for the precise time of the survey. Landsat 5 TM is comprised of seven spectral bands, six of which have a 30 m spatial resolution (3 visible, near infrared (NIR), 2 shortwave infrared (SWIR)), and one with a 120 m spatial resolution [thermal infrared (TIR)] which was resampled to 30 m by the United States Geological Survey (USGS) prior to being download from the USGS Earth Explorer (http://earthexplorer.usgs.gov). From this the Enhanced Vegetation Index (EVI), Land Surface Temperature (LST) and a Soil Moisture Index (SMI) at 30 m spatial resolution were calculated as detailed in Table 1. *A priori*, EVI, LST, SMI and band 7 (SWIR with wavelength 2.08–2.35 μm) were considered to be the variables that were most likely to be associated with tsetse presence and abundance [10, 16]. The values of the four environmental measures were obtained at each trap location, in addition to the mean, minimum, maximum and standard deviation values within a 350 m radius, which corresponds approximately to the estimated daily dispersal rate of male *G. f. fuscipes* [17].

**Table 1:** Summary of environmental data considered when fitting binary logistic and negative binomial geostatistical models to tsetse catch data

Environmental variable	Trap data range	Source	Spatial resolution (m)	Time period	Derivation
Enhanced vegetation index (EVI)	0.07–0.19	Landsat 5	30	December 2009	$\left\{\frac{NIR- Red}{NIR+6\times RED-7.5\times BLUE+1}\right\}$
Soil Moisture Index (SMI)	0.33–0.64	Landsat 5	30	December 2009	$\frac{Tsmax- Ts}{Tsmax- Ts min}$, where Tsmax and Tsmin are the maximum and minimum surface temperatures for a given Normalised Difference Vegetation Index (NDVI) value. See Wang et al. [38]
At-satellite brightness temperature (°C)	25.1–28.9	Landsat 5	30	December 2009	Thermal Infrared Sensor (TIRS) band i.e. Band 6
Land surface temperature (LST)	27.9–34.5	Landsat 5	30	December 2009	At-satellite brightness temperature and NDVI were used to derive LST. Details on the algorithms used can be found in Ndossi et al. [39]
Elevation (m)	852–1210	Shuttle Radar Topography Mission (SRTM)	30	2000	SRTM Void Filled data
Slope (°)	0–7.8	Shuttle Radar Topography Mission (SRTM)	30	2000	Derived from SRTM elevation data using the hydrology tools within the Spatial Analyst Toolbox of ArcGIS (version 10.3.1)
Flow accumulation	0–1451	Shuttle Radar Topography Mission (SRTM)	30	2000	Derived from SRTM elevation data using the hydrology tools within the Spatial Analyst Toolbox of ArcGIS (version 10.3.1)
Fragmentation indices	Various	Advanced Spacebourne Thermal Emission and Reflection Radiometer (ASTER)	15	December 2010	Calculate Normalised Difference Vegetation Index, i.e. $NDVI=\frac{NIR- Red}{NIR+ Red}$ and using a threshold of 0, designate pixels as either vegetated or not vegetated. The R package SDMTools was then used to derive the following patch statistics within 350 m of the trap: • Average distance between patches • Maximum distance between patches • Number of patches • Area covered by patches • Size of largest patch

River data were obtained from the Government of Uganda 1:50,000 maps, which were then converted into shapefiles with each section of river being coded as either main, minor, tertiary, tributary. Further hydrological information was derived from 30 m resolution digital elevation model (DEM) data obtained from the Shuttle Radar Topography Mission (SRTM), accessed *via* the USGS Earth Explorer, i.e. flow direction and flow accumulation as proxy river data. To reflect the situation where there were no accurate river data available for the study area, the proxy river data was derived independently of the river network data that were digitised from published maps. A trial-and-error approach was used to determine a suitable flow accumulation threshold above which it was assumed a river was present. High resolution commercial imagery accessed *via* Google Earth and Bing was used as reference data for this approach. From this a proxy river network was derived and this network was categorised using the Strahler stream order. The raw flow accumulation values (mean, min, max, total) within 350 m of a trap plus the proportion of cells within 350 m of a trap that exceeded a range of flow accumulation thresholds were then calculated, in addition to the distance between the traps and the resulting proxy river network.

Habitat fragmentation measures were based on vegetation values derived at a 15 m resolution using ASTER data, accessed using the USGS Earth Explorer for December 2010. As ASTER data do not include a blue spectral band, it was not possible to derive EVI, hence NDVI (Table 1) was used to identify vegetated from non-vegetated cells, using a threshold of zero. From this, fragmentation indices for the surrounding landscape were calculated, including the average nearest patch distance, total number of vegetated cells and maximum patch size within a 350 m radius of each trap [18–20].

Summaries of the environmental data used in this analysis, and the range of each variable covered by the 236 trap locations are shown in Table 1.

### Statistical analysis

An initial exploratory data analysis was undertaken to determine the likely form of the association between the environmental variables under consideration and fly abundance (average number of flies per trap per day), consisting of scatterplots and simple correlation calculations. Following this, a generalised linear geostatistical model (GLGM) was developed for identifying significant relationships between the total number of tsetse caught in each trap and the environmental covariates. The number of days each trap was operated was included as an offset in the model. The geostatistical modelling framework accounts for the presence of spatial dependency in the trap count data which was not explained by the available environmental variables. Importantly, whilst the model was developed for count data, the application of the model was not to enable precise predictions of fly abundance, but rather to differentiate areas of high and low abundance. As such, an additional predicted outcome of interest was in the form of a relative abundance category as opposed to the exact abundance.

To account for a frequently encountered scenario of there being a lack of reliable local information on the location of the river networks, two forms of the model were considered: one which considered the inclusion of data derived from the Government of Uganda maps (GLGM-River), and one which considered the inclusion of the flow accumulation and proxy river network data derived from the DEM data (GLGM-Proxy). Model fitting was undertaken using an integrated nested Laplace approximation (INLA) in R (version 3.3.1) using the *R-INLA* package (www.r-inla.org) [21–23] and a stochastic partial differential equation (SPDE) approach [21, 24]. Within the *R-INLA* package we considered modelling the count data using Poisson and negative binomial distributions and their respective type 1 zero-inflated versions to account for the excess number of traps were no flies were caught. Details of these can be found within the *R-INLA* documentation [25].

A systematic approach was adopted to model fitting with the aim of determining a parsimonious model capable of predicting fly abundance. First, the Pearson’s correlation between each of the continuous variables was calculated, and where the correlation exceeded 0.7, only one of the variables was selected to be considered in the model. Following this, the potential set of covariates was reduced further by fitting a regression model to the full data set and applying a Lasso (Least Absolute Shrinkage and Selection Operator) penalty. INLA was then used to fit the GLGMs to the data, which each included a single environmental variable. Where appropriate, various transformations (e.g. square root, log-transformed) of, and interactions between, the environmental variables were considered to account for non-linear relationships between the environment and the outcome. For each fitted model, the Watanabe-Akaike information criterion (WAIC) was obtained, allowing the relative fit of each model to be compared [26]. Models were then ranked according to their WAIC, with smaller values indicating better predictive values. Starting with the model with the smallest WAIC, environmental covariates were added to the model one at a time according to their rank order. Initially a zero-inflated negative binomial was used during the model selection process. The WAIC was then used to compare this to less complex negative binomial and Poisson models, plus make a comparison between models with and without a spatial term.

The performance of the models was then assessed with respect to their goodness of fit, both absolutely and within fly abundance categories. Using R-INLA, 1000 samples were drawn from the posterior distributions of the fitted models (see Additional file 2) to enable summary measures of the fitted values to be produced. Firstly, the root mean square error of the fitted model was assessed using the posterior mean as the fitted model estimate of fly catches. In addition, the fitted probability *p*_*ij*_ that the fly count at trap *i* was within the range of the observed count category *j* was obtained. In practice, this was obtained using the proportion of the 1000 posterior samples that were within each category. Violin plots of *p*_*ic*_ by observed category were then produced, where *p*_*ic*_ is the fitted probability that trap *i* was within the range of the correct observed count category *c*. Categories considered were based on terciles of the catch data (‘low’, ‘medium’, ‘high’), plus a range of binary categories were also considered. Using a threshold based on the average number of flies caught per trap per day to divide the data into two categories, the Brier score [27] and receiver-operating characteristic (ROC) curve [28] were obtained for the fitted models as a measure of how well the model was able to discriminate between the categories. The Brier score is the sum of the squared difference between the observed outcome *y*_*i*_, which is equal to 1 if the binary threshold is exceeded and zero otherwise, and the fitted probability *p*_*i*_ that the fly count at trap *i* exceeded the binary threshold, i.e. ${\sum}_i{\left({y}_i-{p}_i\right)}^2$. The ROC curve is a plot of the sensitivity against false positive rate for a range of probability thresholds, assuming that the observed data are the true state of nature. From this curve it is possible to calculate the area under the curve (AUC) as a measure of how well the model is able to discriminate between the two categories. Both measures range between zero and one, with a Brier score of 0 and a ROC-AUC of 1 representing perfect discrimination.

Maps of estimated tsetse abundance were produced, with estimates being made on a 30 m by 30 m grid using functions within the *R-INLA* package (see Additional file 2: S2). Maps included the posterior mean fly abundance and the probability of exceeding relevant category thresholds. To disentangle the impact of the environmental covariates and the residual spatial correlation on the predictions a separate map of the latter was also produced.

## Results

### Exploratory data analysis

**Fig. 2:**
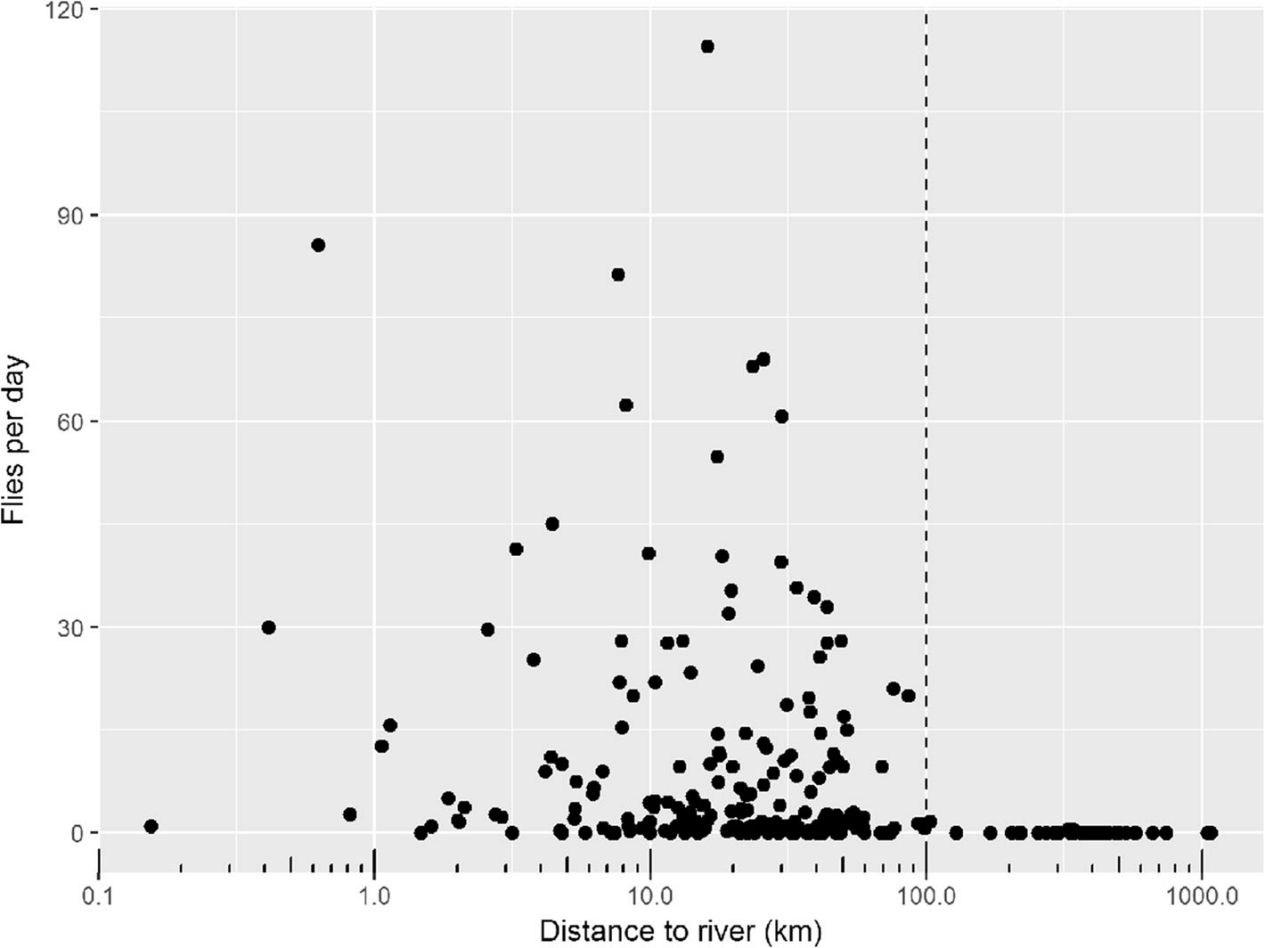
A plot of average daily catch against Euclidean distance to the nearest river on the log_10_ scale. Traps within 100 m of the river (denoted by a dashed vertical line) were used to develop a geostatistical model of tsetse abundance

Of the 236 traps that were monitored during the baseline survey, 64 traps (27%) did not catch any tsetse (Fig. 1). On reflecting on the initial study design, it was noted that the vast majority of traps which were placed in locations far from the river network rarely caught any flies (Fig. 2) in accordance with the expected distribution of riverine tsetse. Due to this observation, and the additional knowledge that current control interventions are focused on deploying “Tiny Targets” along river networks, the decision was made to focus only on modelling the variability in tsetse catches within close proximity to the rivers. Using an arbitrary distance threshold of 100 m, this resulted in data from 198 traps being used to develop the abundance models. Of these 198 traps, 15% traps (29/198) did not catch any flies, whereas in the remainder the fly abundance ranged from 0.3 to 114.5 flies per day with a median of 3.7.

**Fig. 3:**
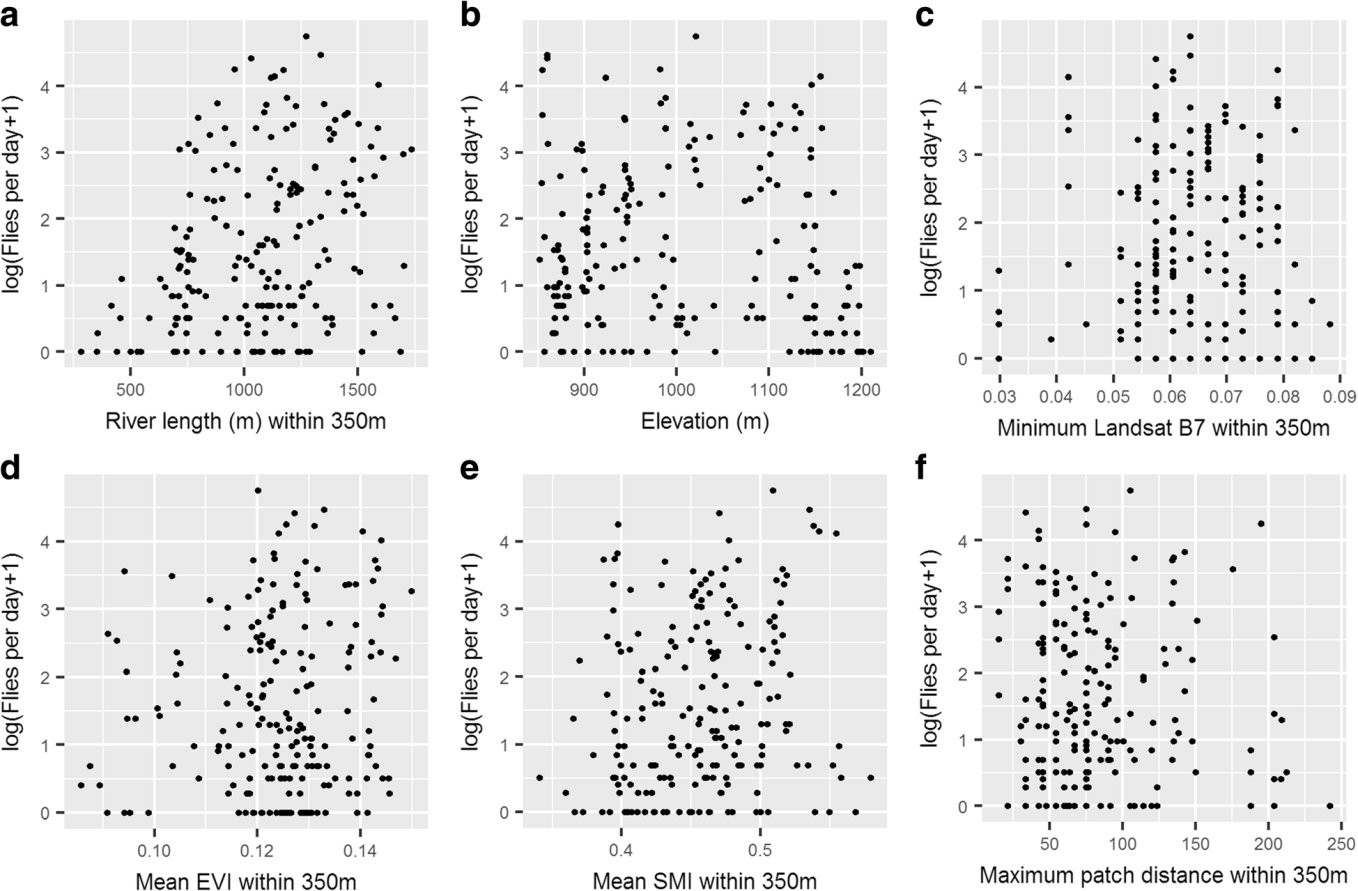
Scatter plots of environmental variables against the log-transformed average daily tsetse count at each of the 198 trap locations: (**a**) river length within 350 m; (**b**) elevation in metres; (**c**) minimum value of Landsat band 7 within 350 m; (**d**) mean EVI within 350 m; (**e**) mean SMI within 350 m; (**f**) maximum distance between vegetated patches within 350 m

Simple scatterplots were initially produced of the log-transformed average fly count (plus one, to avoid errors resulting from log-transforming zero) and the extracted environmental variables. Figure 3 presents a subset of these plots. There appeared to positive relationships between log-transformed catch and distance to the river plus the length of river within a 350 m radius, however the relationship with other variables was less clear.

**Fig. 4:**
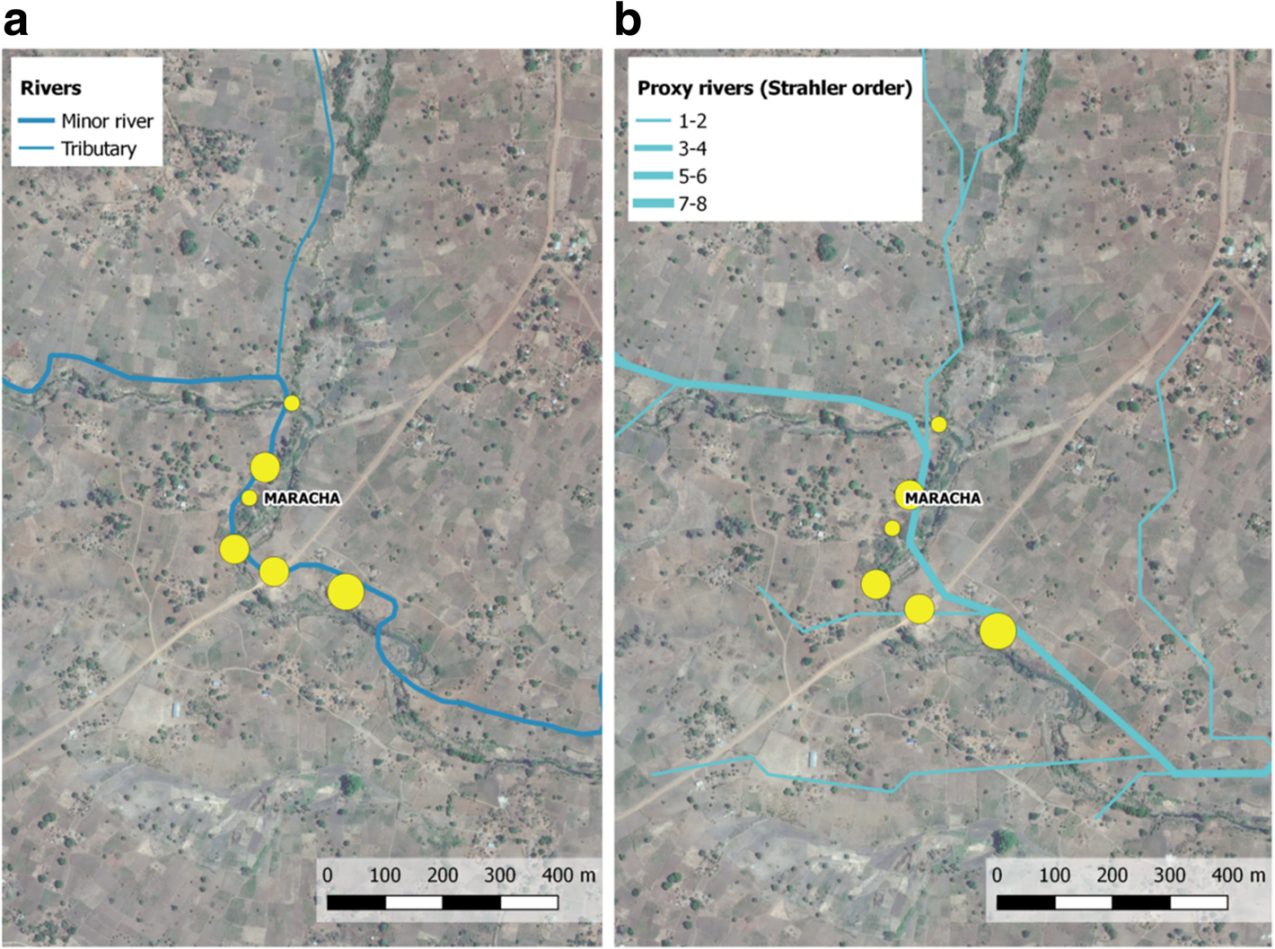
Comparison of river network data obtained from the Government of Uganda (**a**) and derived from 30 m resolution digital elevation data using a flow accumulation threshold of 100 (**b**)

With regards to alternative sources of river data, after visually examining the relationship between flow accumulation and visible river networks in Google satellite imagery, proxy river shapefiles were derived using a flow accumulation threshold of 100. There was a relatively weak significant relationship between the distances between the rivers and the traps obtained using the two river sources (Spearman’s correlation, *r* = 0.35, *P* ≤ 0.0001), indicating that whilst the network obtained using the flow accumulation can give the approximate location of rivers in an area, this is not an overly precise way of identifying river paths at a small geographical scale (Fig. 4) due to the resolution of the digital elevation data being used (30 m).

### Zero-inflated negative binomial models

Given the relatively large number of zeros in the dataset, plus evidence of additional overdispersion provided by several traps having very large catches (> 100 tsetse per day), a zero-inflated negative binomial (ZINB) geostatistical modelling approach was initially adopted. Following the systematic model fitting procedure, the variables included in the final ZINBGM-River model (Table 2) were distance to nearest river (in metres), excluding those classed as tributaries, elevation, log-transformed length of river within 350 m, and the minimum value of Landsat band 7 within 350 m, with river distance and elevation having a negative relationship with fly abundance, and river length and band 7 having a positive relationship. Similarly, the ZINBGM-Proxy model included a negative association with elevation and a positive association with a proxy measure of the proportion of the surrounding area covered by ‘large’ rivers, i.e. the square root of the proportion of surrounding cells (within 350 m) with flow accumulation greater than 2000 and the minimum value of Landsat band 7 within 350 m. In both instances the zero-inflated term was modelled using an intercept only had a lower WAIC (river model = 1454, proxy model = 1464) than both the negative binomial with no zero-inflated component (river model = 1465, proxy model = 1474) and a zero-inflated Poisson model (river model = 2353, proxy model = 2869). The WAIC for both zero-inflated models without a spatial component was also substantially larger (river model = 1562, proxy model = 1554) indicating that after adjusting for the effects of the covariates there was still significant spatially structured variability in the data.

**Table 2:** Results of the fitted ZINBGM-River and ZINBGM-Proxy models

Model	Covariates	Posterior mean	95% credible interval	WAIC
ZINBGM-River	Distance to nearest river (excluding tributaries)	-0.0024	-0.0033, -0.0014	1454
	Elevation	-0.0060	-0.0143, 0.0007	
	Log (river length) within 350 m	0.9808	0.2556, 1.7092	
	Min (Band 7) within 350 m	15.54	-5.44, 36.32	
ZINBGM-Proxy	Elevation	-0.0060	-0.0142, 0.0002	1464
	Sqrt (proportion with flow accumulation > 2000) within 350 m	10.96	7.61, 14.24	
	Min (Band 7) within 350 m	23.12	2.08, 43.87	

### Model evaluation

**Fig. 5:**
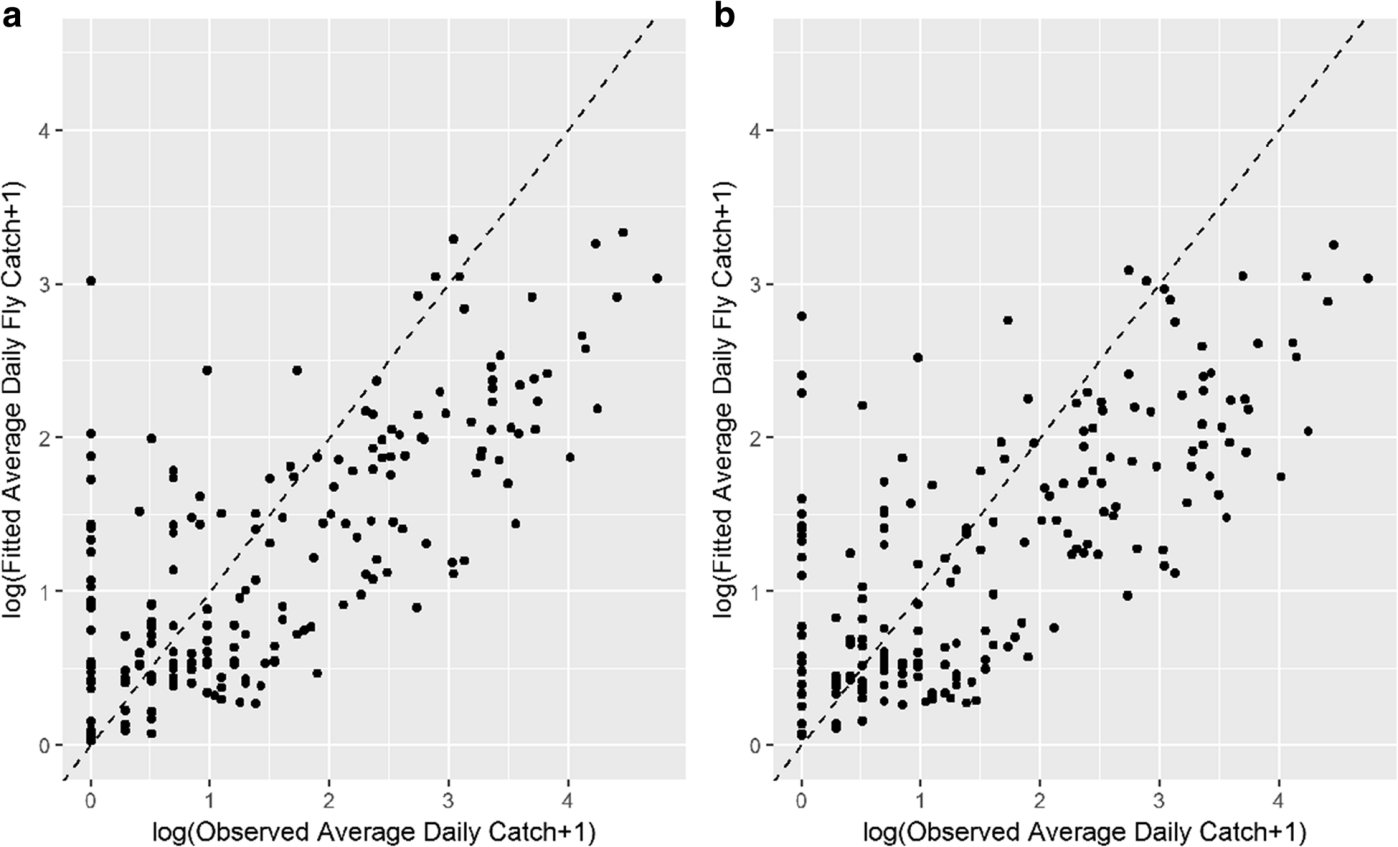
Scatter plots of observed average daily tsetse count against mean fitted and the posterior mean daily tsetse count on the log scale obtained using data from the 198 traps for the ZINBGM-River model (**a**) and the ZINBGM-Proxy model (**b**)

**Fig. 6:**
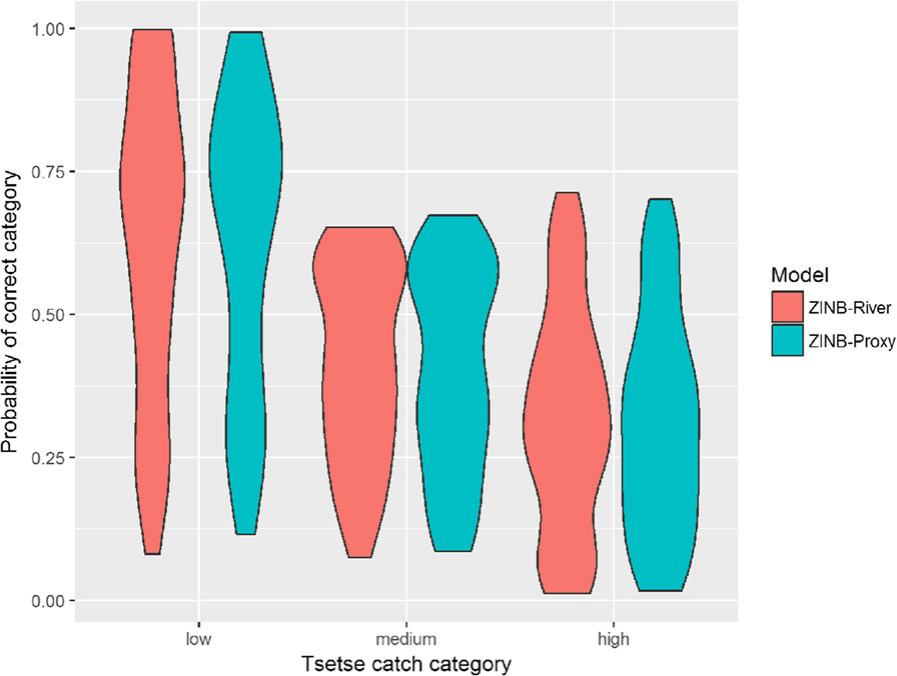
Violin plots depicting the distribution of the posterior probability that the daily fly count was within the correct observed range of low, medium and high for the ZINBGM-River model (**a**) and the ZINBGM-Proxy model (**b**)

**Fig. 7:**
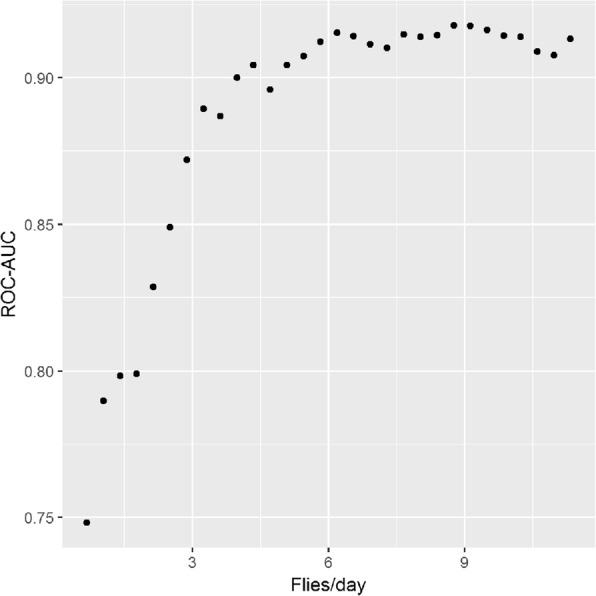
Performance of the ZINBGM-River model with respect to categorising the traps into two categories-based thresholds ranging from 0.667 flies/day to 11.667 flies per day. Performance is measured using the ROC-AUC measure

Figure 5 presents a scatterplot of the observed average daily catch against the posterior mean average daily catch on the log scale for both models. Despite accounting for excess zeros in the model, the model still over-estimates the fly counts when the observed numbers are low but appears to under-predict when counts are high. The RMSE for the ZINBGM-River model was 15.2, in comparison to 15.4 for the ZINBGM-Proxy model. With regards to the ability of the model to categorise the fly counts correctly, Fig. 6 presents the violin plots of the fitted *p*_*ic*_, i.e. the proportion of posterior samples for trap *i* that were within the correct category *c*, *c* = 1, 2, 3 where categories were based on the terciles of the trap data, i.e. low = [0, 1), medium = [1, 7.67), high = [7.67, 114]. Whilst the fitted probability of counts correctly falling in the ‘low’ category is generally high (median = 0.70 for both models), neither model is able to well distinguish between the medium and high categories. In reducing the data to two categories using an initial threshold of one fly per day, the ROC-AUC and Brier scores for the two models were similar (ROC_AUC: ZINB-River = 0.7869, ZINB-Proxy = 0.7633; Brier: ZINB-River = 0.1949, ZINB-Proxy = 0.2015), with optimal thresholds for simultaneously maximising sensitivity and specificity of 0.54 and 0.60 for the two models, respectively. In considering a range of binary categories with thresholds ranging from the first quartile of the observed data (0.667 flies/day) to the third quartile (11.3 flies/day), the model performs best at distinguishing between areas with ‘moderate’ fly relative abundance. Figure 7 presents a plot of the ROC-AUC obtained using a range of categorisation thresholds for the ZINB-River model, depicting that the ROC-AUC exceeds 0.9 when the catch threshold exceeds 5 flies/day.

**Fig. 8:**
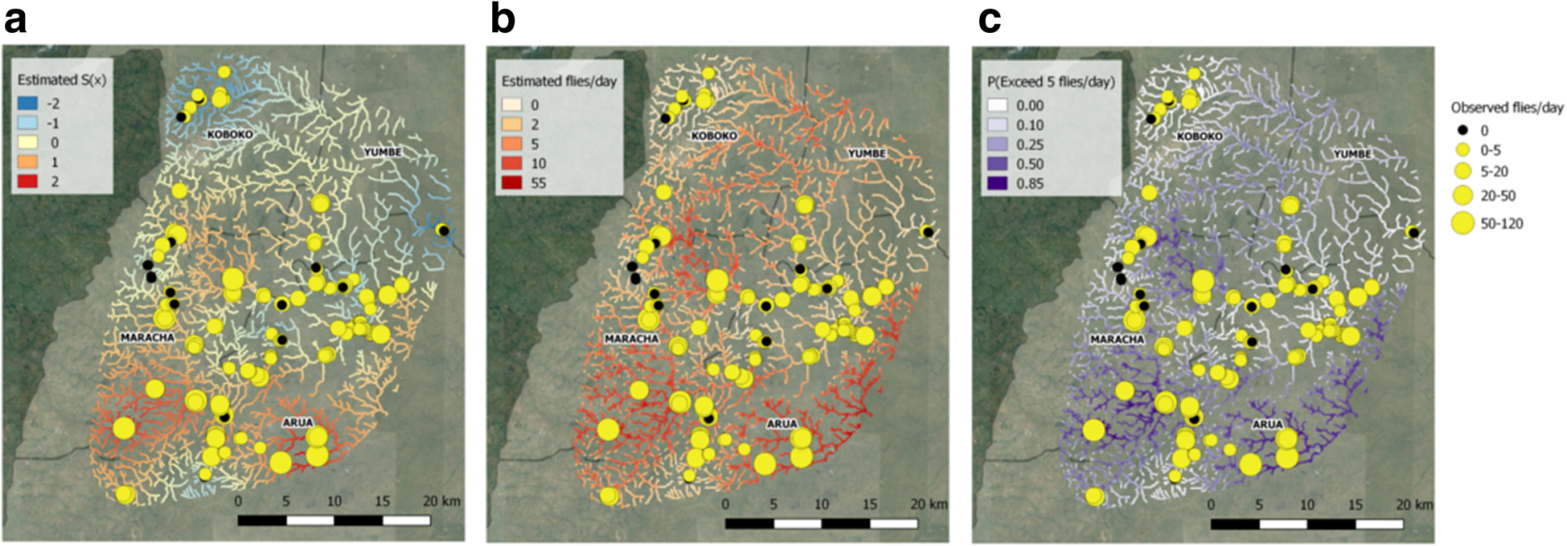
Maps of the spatial term obtained from fitting the ZINBGM-River model (**a**), the estimated catch per day (**b**) and probability of exceeding the threshold of five flies/day (**c**)

Figure 8 presents maps of the fitted spatial process obtained using the ZINBGM-River model, the posterior predictive mean number of flies per day, and the associated probability of exceeding five flies per day for areas within 100 m of the river network. After accounting for the variability in the selected environmental risk factors, it was observed that there were areas of higher than expected fly counts in the southern part of the study region, whereas lower than expected counts were observed in the north-east and the north-west (Fig. 8a). Figure 8b and c indicated that large numbers of flies were expected, both in terms of the posterior mean of the predicted counts and in the predicted probability that the number of flies exceeded five per day, in the south east and south west of the study region.

## Discussion

In this paper we consider estimating the relative abundance of tsetse using a zero-inflated negative binomial geostatistical modelling approach. Two sources of river network data were considered when fitting these models to account for scenarios where accurate locally-obtained river network information was available. Following a systematic model fitting approach, both types of model shared common environmental covariates including elevation, a measure of soil/vegetation moisture (Landsat 5 Band 7) and a measure of how much river water was in the surrounding area. As anticipated, given that the area was inhabited by riverine tsetse species, there was a negative relationship between the distance to larger rivers (i.e. excluding tributaries) and the relative abundance of tsetse using the more accurate locally-obtained river network data. It has long been recognised that *G. f. fuscipes* is found in more humid habitats in comparison to other subspecies (*G. f. quanzensis*, *G. f. martini*) [29] and laboratory studies have also shown that in comparison to six other species of tsetse, *G. f. fuscipes* was the least tolerant of arid environments [30].

Proxy river data derived using digital elevation data (30 m resolution) could assist in identify the general location of rivers, including the ‘amount’ of water within an area, but it could not be used to estimate the precise distance between traps and the river at a small spatial scale (< 100 m). This result highlights the need to obtain as accurate water network data as possible when developing local-scale models for riverine tsetse. Other remotely sensed-based methods in addition to those employed in this paper are available to identify river networks, such as those based on land cover/land use classification [31, 32]. Given that the rivers in this area were generally quite narrow we however found that the spatial resolution of publicly available contemporary remote sensing data such as Landsat was inadequate for this task. As more remote sensing resources become available, for example Sentinel-2 launched in June 2015 collects data with a spatial resolution of 10 m, more accurate river network information will be increasingly accessible.

Landsat 5 data from a single time point in December 2009, i.e. one year prior to the collection of the tsetse count data were used to conduct this analysis, as contemporary data were not available due to cloud cover. Whilst this is a limitation in this analysis, the authors note that in areas where no “Tiny Targets” were deployed there was little observed seasonal or inter-annual variability in tsetse catches [4]. As such, it was feasible to use relatively recent satellite imagery to explore the relationship between the environment and tsetse catches.

The analysis conducted in this paper focused on explaining the variability in tsetse catches within close proximity to rivers (as identified using Government of Uganda maps). This decision was driven by the observation that flies were rarely found in sampling sites away from the river network during the sampling period. Whilst this was an appropriate decision to make given the available data, it is important to acknowledge the importance of verifying the spatial extent of riverine tsetse habitat when surveying new areas, as tsetse may be more dispersed elsewhere.

Previous research into mapping tsetse has focused on mapping the probability of tsetse presence either using presence/absence data and approaches such as logistic regression and discriminant analysis, or presence only data using approaches such as MaxEnt [10, 33, 34]. Whilst this approach is useful in identifying general areas where tsetse control is appropriate, in settings such as northern Uganda where tsetse densities are generally high, there is a greater interest in gaining an understanding of fly density variability for the purposes of guiding and prioritising control efforts. To the authors knowledge, this is the first paper to develop maps of estimated abundance of *T. b. gambiense*. Whilst the efficiency of the pyramidal traps used in this study are affected by a number of factors in addition to the surrounding fly density, the presented zero-inflated negative binomial models were still able to detect trends in the number of flies caught and the surrounding environment, and enabled areas with high *versus* low fly numbers to be differentiated. Such information could assist in the planning and implementation of tsetse control operations in several respects. First, it provides a fine-scale map of the likely distribution and abundance of tsetse which can guide where targets should be deployed and the required frequency of the deployment [35]. Secondly, the identification of areas where tsetse are predicted to be abundant and hence serve as sites for entomological monitoring of control operations. Thirdly, improved knowledge of the local distribution and abundance will also assist in the identification of sites where disease risk is greater. These ‘hotspots’ may warrant increased monitoring of the human and vector populations and/or enhanced levels of vector control. Finally, by quantifying relationships between environmental variables and tsetse we have the basis of a method to predict the likely impact of changes in land-use on tsetse and tsetse-borne diseases.

The data used to develop these models were collected over a short time period during the dry season, over a relatively small area with limited environmental variability. With the aim of expanding the control of tsetse over larger geographical areas, the focus is now on developing these models further to ensure that they remain valid for larger geographical areas where tsetse control has not yet been implemented in northern Uganda, South Sudan and eastern parts of the Democratic Republic of Congo where *G. f. fuscipes* spreads Gambian sleeping sickness. A similar approach has also been adopted to predict the distribution and abundance of savanna species of tsetse in Tanzania [36].

## Conclusions

There is a growing body of evidence that vector control can make a valuable contribution to the control Gambian sleeping sickness in addition to active and passive screening and treatment of the human population [37]. Vector control relies on identifying areas where tsetse are present so that the vector control can be applied cost-effectively. Analysis of the relationship between remotely sensed environmental variables and the numbers of riverine tsetse, i.e. *G. f. fuscipes* caught from traps, showed that we were able to predict daily average fly catches, and differentiate between areas of high and low fly counts. These models can now be used to assist in the design, implementation and monitoring of tsetse control operations to eliminate Gambian HAT in northern Uganda and further can be used as a framework by which to undertake similar studies in other areas where *G. f. fuscipes* spreads Gambian sleeping sickness.

## Additional files

Additional file 1: Tsetse data and their associated environmental covariates at each of the 236 baseline trapping sites. (CSV 48 kb)

Additional file 2: R code demonstrating the use of INLA to fit zero-inflated negative binomial geostatistical models to the tsetse count data. (R 4 kb)

## Abbreviations

AUC: area under the curve
HAT: Human African trypanosomiasis
INLA: Integrated Nested Laplace Approximation
RMSE: root mean squared error
ROC: receiver-operating curve
